# Draft Genome Sequence of Lactobacillus brevis Strain 2-34, Isolated from the Shaoxing Huangjiu Fermentation Process

**DOI:** 10.1128/mra.00710-22

**Published:** 2022-09-12

**Authors:** Xuemei Li, Chenghao Li, Qili Mi, Gaofeng Dong, Qian Gao, Xiaomin Li

**Affiliations:** a Yunnan Key Laboratory of Tobacco Chemistry, China Tobacco Yunnan Industrial Co., Ltd., Kunming, China; b National Engineering Research Center for Cereal Fermentation and Food Biomanufacturing, Jiangnan University, Wuxi, China; University of Southern California

## Abstract

Here, we announce the draft genome sequence of Lactobacillus brevis 2-34, a strain isolated from the fermentation process of Shaoxing huangjiu (Chinese rice wine). The genome size of 2-34 was 2,557,496 bp, with 2,459 coding genes, 67 tRNAs, and 2 rRNAs.

## ANNOUNCEMENT

Lactobacillus brevis strain 2-34 was isolated with several other lactic acids from fermentation liquor of Shaoxing huangjiu (Chinese rice wine) (1). This strain shows a remarkable ability to reuptake and assimilate citrulline (2), one of the main precursors of the carcinogenic compound ethyl carbamate (EC) in Shaoxing huangjiu (3). Strain 2-34 has great potential for use in Chinese rice wine fermentation to eliminate citrulline and therefore control the EC content in the final product.

The Chinese rice wine primary fermentation liquor was obtained from the Guyuelongshan Shaoxing Huangjiu Co. (Shaoxing, Zhejiang, China). Serial dilutions were made in 0.9% sodium chloride and plated onto de Man-Rogosa-Sharpe (MRS) agar. After incubation at 30°C for 48 h under anaerobic conditions, the isolated colonies were grown anaerobically in 10 mL MRS broth at 30°C for 24 h. A single colony of strain 2-34 was picked for culturing prior to DNA isolation. Genomic DNA was extracted using the rapid bacterial genomic DNA isolation kit (Sangon Biotech, Shanghai, China) according to the manufacturer’s instructions and then submitted to Majorbio (Shanghai, China) for library preparation using the NGS fast DNA library prep set for Illumina (Bjbiolab, Beijing, China) after Covaris shearing. The whole genome was sequenced using an Illumina HiSeq X Ten platform with the 2 × 150-bp paired-end sequencing protocol. The 8,731,560 raw reads produced were then trimmed using Trimmomatic v0.39 (4) and *de novo* assembled using SOAPdenovo2 v2.04 (5), generating 87 contigs with a genome length of 2,557,496 bp, 120-fold coverage, a GC content of 45.71%, and an *N*_50_ value of 71,034 bp. Annotation using PGAP v6.1 (6) predicted 2,614 total genes, 2,459 coding genes, 67 tRNAs, and 2 rRNAs.

The L. brevis 2-34 genome was assessed for the presence of clustered regularly interspaced short palindromic repeat (CRISPR) arrays using CRISPRFinder (7), and 6 were detected, located on scaffolds 1, 19, 28, 30, 48, and 58. In addition, genomic islands (three, located on scaffolds 14, 25, and 41) and prophages (two intact and one incomplete, located on scaffolds 19, 21, and 31) were detected using the integrative online tools IslandViewer 4 (8) and PHASTER (9), respectively. Furthermore, a cluster consisting of 17 bacteriocin genes was detected in scaffold 21 (approximately 37845 to 48721 bp) using BAGEL4 (10). No genes encoding potential virulence or pathogenicity factors were detected. Default parameters were used for all software unless otherwise specified.

The draft genome sequence of L. brevis 2-34 will be useful for further studies of specific gene features and for understanding its mechanisms for citrulline reuptake in the huangjiu fermentation system.

### Data availability.

All data are available at GenBank under BioProject accession number PRJNA857749. The GenBank accession number is JANDJV000000000. The version described in this paper is the first version. The BioSample accession number is SAMN29636078.

## References

[1] Yu W, Li X, Lu J, Xie G. 2018. Citrulline production by lactic acid bacteria in Chinese rice wine. J Inst Brew 124:85–90. doi:10.1002/jib.475.

[2] Yu W, Xie G, Wu D, Li X, Lu J. 2020. A *Lactobacillus brevis* strain with citrulline re-uptake activity for citrulline and ethyl carbamate control during Chinese rice wine fermentation. Food Biosci 36:100612. doi:10.1016/j.fbio.2020.100612.

[3] Zhao X, Zou H, Fu J, Zhou J, Du G, Chen J. 2014. Metabolic engineering of the regulators in nitrogen catabolite repression to reduce the production of ethyl carbamate in a model rice wine system. Appl Environ Microbiol 80:392–398. doi:10.1128/AEM.03055-13. 24185848 PMC3910993

[4] Bolger AM, Lohse M, Usadel B. 2014. Trimmomatic: a flexible trimmer for Illumina sequence data. Bioinformatics 30:2114–2120. doi:10.1093/bioinformatics/btu170. 24695404 PMC4103590

[5] Luo R, Liu B, Xie Y, Li Z, Huang W, Yuan J, He G, Chen Y, Pan Q, Liu Y, Tang J, Wu G, Zhang H, Shi Y, Liu Y, Yu C, Wang B, Lu Y, Han C, Cheung DW, Yiu S-M, Peng S, Xiaoqian Z, Liu G, Liao X, Li Y, Yang H, Wang J, Lam T-W, Wang J. 2012. SOAPdenovo2: an empirically improved memory-efficient short-read de novo assembler. Gigascience 1:18. doi:10.1186/2047-217X-1-18. 23587118 PMC3626529

[6] Tatusova T, DiCuccio M, Badretdin A, Chetvernin V, Nawrocki EP, Zaslavsky L, Lomsadze A, Pruitt KD, Borodovsky M, Ostell J. 2016. NCBI Prokaryotic Genome Annotation Pipeline. Nucleic Acids Res 44:6614–6624. doi:10.1093/nar/gkw569. 27342282 PMC5001611

[7] Grissa I, Vergnaud G, Pourcel C. 2007. CRISPRFinder: a Web tool to identify clustered regularly interspaced short palindromic repeats. Nucleic Acids Res 35:W52–W57. doi:10.1093/nar/gkm360. 17537822 PMC1933234

[8] Bertelli C, Laird MR, Williams KP, Lau BY, Hoad G, Winsor GL, Brinkman FSL, Simon Fraser University Research Computing Group. 2017. IslandViewer 4: expanded prediction of genomic islands for larger-scale datasets. Nucleic Acids Res 45:W30–W35. doi:10.1093/nar/gkx343. 28472413 PMC5570257

[9] Arndt D, Grant JR, Marcu A, Sajed T, Pon A, Liang Y, Wishart DS. 2016. PHASTER: a better, faster version of the PHAST phage search tool. Nucleic Acids Res 44:W16–W21. doi:10.1093/nar/gkw387. 27141966 PMC4987931

[10] van Heel AJ, de Jong A, Song C, Viel JH, Kok J, Kuipers OP. 2018. BAGEL4: a user-friendly Web server to thoroughly mine RiPPs and bacteriocins. Nucleic Acids Res 46:W278–W281. doi:10.1093/nar/gky383. 29788290 PMC6030817

